# Anti-Melanogenic and Antioxidant Effects of Cell-Free Supernatant from *Lactobacillus gasseri* BNR17

**DOI:** 10.3390/microorganisms10040788

**Published:** 2022-04-08

**Authors:** Sol Lee, Han-Oh Park, Wonbeak Yoo

**Affiliations:** 1 AceBiome Inc., Seoul 06164, Korea; slee@acebiome.com (S.L.); hpark@bioneer.co.kr (H.-O.P.); 2 R&D Center, AceBiome Inc., Daejeon 34013, Korea; 3 siRNAgen Therapeutics, Daejeon 34302, Korea; 4 Bioneer Corporation, Daejeon 34302, Korea

**Keywords:** cell-free supernatant, *Lactobacillus gasseri* BNR17, anti-melanogenesis, antioxidative effect

## Abstract

In recent years, there has been considerable interest in the use of cell-free supernatant of probiotics culture for nutritional and functional applications. In this study, we investigated the effect of the cell-free supernatant from *Lactobacillus gasseri* BNR17 (CFS) on anti-melanogenesis and reducing oxidative stress in B16-F10 murine melanoma cells and HaCaT human keratinocytes. Treatment with CFS significantly inhibited the production of extracellular and intracellular melanin without cytotoxicity during melanogenesis induced by the α-MSH in B16-F10 cells. The CFS dramatically reduced tyrosinase activity and the melanogenesis-related gene expression. Further, it showed antioxidative effects in a dose-dependent manner in DPPH (2,2-diphenyl-1-picryl-hydrazyl-hydrate) assays and significantly increased the mRNA levels of *HO-1* and *CAT* in HaCaT cells. Furthermore, the CFS increased HO-1 and anti-oxidative-related gene expression during H_2_O_2_-induced oxidative stress in HaCaT cells. Together, this study suggests that the CFS reduces hyperpigmentation and inhibits oxidative stress, and thus can be used as a potential skincare product in the future.

## 1. Introduction

Melanin is the main component of skin color and protects the skin against harmful UV radiation damage under physiological conditions [1]. However, abnormal melanin production can result in hyper pigmentary disorders such as freckles, skin discoloration, melasma, moles, and lentigo [2]. In mammals, melanin biosynthesis is controlled by enzymes such as tyrosinase (TYR). Microphthalmia-associated transcription factor (MITF), tyrosinase-related protein 1 (TYRP1), and tyrosinase-related protein 2 (TYRP2) also contribute to melanin production. Specifically, melanin is synthesized from L-tyrosine by the action of tyrosinase. L-tyrosine is converted to dihydroxyphenylalanine (L-DOPA) and then to DOPA quinone, a precursor of melanin [3,4]. Additionally, melanogenesis produces hydrogen peroxide (H_2_O_2_) and other reactive oxygen species (ROS) that expose human melanocytes to high levels of oxidative stress [5].

Skin is constantly exposed to both exogenous and endogenous oxidative stress, resulting in oxidative damages due to excessive ROS and lipid peroxidation. Especially, oxidative stress, including H_2_O_2_ and other ROS, can cause cell death and damage following UV irradiation and produce melanin in melanocytes [6,7]. In the synthesis of melanin, by generating ROS, melanin synthesis cells are placed in an oxidative stress environment, and melanin biosynthesis is continuously induced [8]. Further, oxidative stress is linked to the pathogenesis of skin disorders, such as aging, wrinkles, and skin cancer [9,10,11]. In addition, the increase in UV irradiation-induced ROS increases the expression level of matrix metalloproteinases, which are responsible for increasing collagen degradation [12]. Since inhibition of oxidative stress is favorable for reducing and/or preventing atypical pigmentation in melanocytes, various studies have been conducted to study the development of complex mixtures exhibiting antioxidant effects as well as the inhibition of tyrosinase activity [13,14]. Therefore, antioxidants are important not only for downregulating melanogenesis, but also for maintaining healthy skin.

Oxidative stress, including H_2_O_2_ and other ROS, can cause cell death and damage following UV irradiation and produce melanin [6,7]. Further, oxidative stress is linked to the pathogenesis of skin disorders, such as aging, wrinkles, and skin cancer [9,10,11]. In addition, the increase in UV irradiation-induced ROS increases the expression level of matrix metalloproteinases that inhibit collagen synthesis [12]. Therefore, antioxidants are important not only for downregulating melanogenesis but also for maintaining healthy skin.

*Lactobacillus gasseri* BNR17, a lactic acid bacterium, was isolated from human breast milk [15]. Our previous studies have shown that *L. gasseri* BNR17 has potential benefits in irritable bowel syndrome [16], weight control [17], type 2 diabetes [18], and alleviating menopausal symptoms [19]. However, no studies have assessed the anti-melanogenic and antioxidant activities of the cell-free supernatant from *L. gasseri* BNR17 (CFS). Thus, this study aimed to verify the beneficial effects of CFS on the skin and whether it can be developed as a skincare product in the future.

## 2. Results

### 2.1. Effect of CFS on Cell Viability in B16-F10 and HaCaT Cells

**Figure 1:**
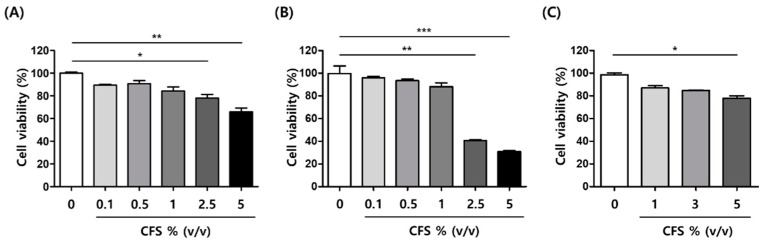
Effect of CFS on B16-F10 and HaCaT cell viability. B16-F10 cells were treated with various concentrations of CFS for (**A**) 24 h and (**B**) 48 h. (**C**) HaCaT cells were treated with various concentrations of CFS for 24 h. Cell viability was assessed by WST-1 assay. CFS: cell-free supernatant from *L. gasseri* BNR17. Values represent the mean ± SD of three independent experiments. * *p* < 0.05, ** *p* < 0.01, and *** *p* < 0.001 for control versus sample.

The cell viability assay was performed to determine whether the CFS was cytotoxic to B16-F10 melanocytes and HaCaT keratinocytes. As shown Figure 1, no significant cytotoxic effect was observed up to 1% (*v*/*v*) CFS in B16-F10 cells (Figure 1A) and up to 3% (*v*/*v*) CFS in HaCaT cells (Figure 1C) for 24 h. Thus, a CFS concentration below 1% (*v*/*v*) in B16-F10 and 3% (*v*/*v*) in HaCaT was used for the subsequent experiments.

### 2.2. CFS Decreases Melanin Synthesis in B16-F10

**Figure 2:**
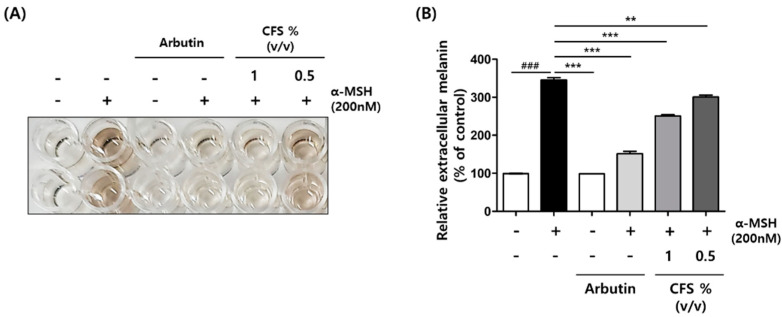
Effect of CFS on melanin secretion in B16-F10 cells. B16-F10 cells were exposed to 200 nM α-MSH in the presence of 0.5 and 1% (*v*/*v*) CFS or 200 µM AB. (**A**) Supernatant color of B16-F10 samples and (**B**) medium melanin contents. The melanin levels were determined as described in the Materials and Methods. CFS: cell-free supernatant from *L. gasseri* BNR17. Values represent the mean ± SD of three independent experiments. ^###^ *p <* 0.001 compared with control. ** *p <* 0.01 and *** *p <* 0.001 compared with the α-MSH-treated control.

To determine the effect of CFS on melanin secretion, B16-F10 cells were treated with various CFS concentrations as shown in Figure 2. By visual observation, 0.5 and 1% (*v*/*v*) CFS significantly inhibited the melanin secretion into the culture medium in alpha-melanocyte-stimulating hormone (α-MSH)-induced B16F0 cells, which was similar to the effect elicited by arbutin (positive control) (Figure 2A,B).

**Figure 3:**
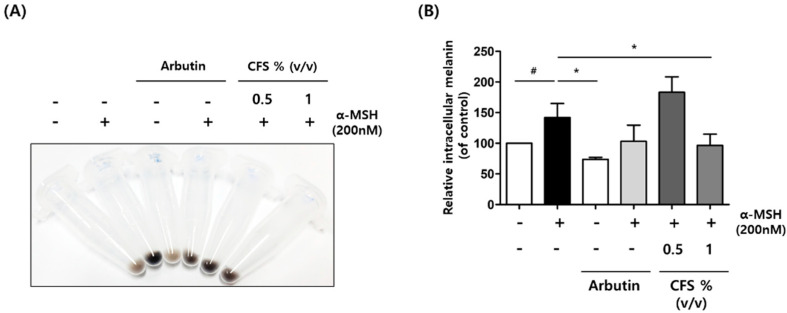
Effect of CFS on melanin production in B16-F10 cells. B16-F10 cells were exposed to 200 nM α-MSH in the presence of 0.5 and 1% (*v*/*v*) CFS or 200 µM AB. (**A**) Representative images of cell pellets after CFS treatment and (**B**) intracellular melanin contents. The melanin levels were determined as described in the Materials and Methods. CFS: cell-free supernatant from *L. gasseri* BNR17. Values represent the mean ± SD of three independent experiments. ^#^ *p* < 0.05 compared with control. * *p* < 0.05 compared with the α-MSH-treated control.

To confirm the effects of CFS on intracellular pigmentation, the intracellular melanin content was measured using the melanin content assay. Similar to the result of arbutin treatment, the color of the CFS-pretreated cells was brighter than that of the only α-MSH-treated cells (Figure 3A). Notably, treatment with 1% (*v*/*v*) CFS significantly reduced the cellular melanin content of α-MSH-stimulated cells (Figure 3B).

In addition, the anti-melanogenic effect of CFS was maintained over a broad temperature range (37 °C and 100 °C) (Figure S1A). Further, the inhibitory effect of the CFS on melanin synthesis exhibited good thermostability for extracellular (Figure S1B) and intracellular melanin (Figure S1C), and the reduction in melanin production by CFS was similar to that of arbutin. These results suggest that CFS exerts an anti-melanogenic effect by reducing melanin synthesis with good thermostability in B16-F10 cells.

### 2.3. CFS Reduced Intracellular Tyrosinase Activity in B16-F10

**Figure 4:**
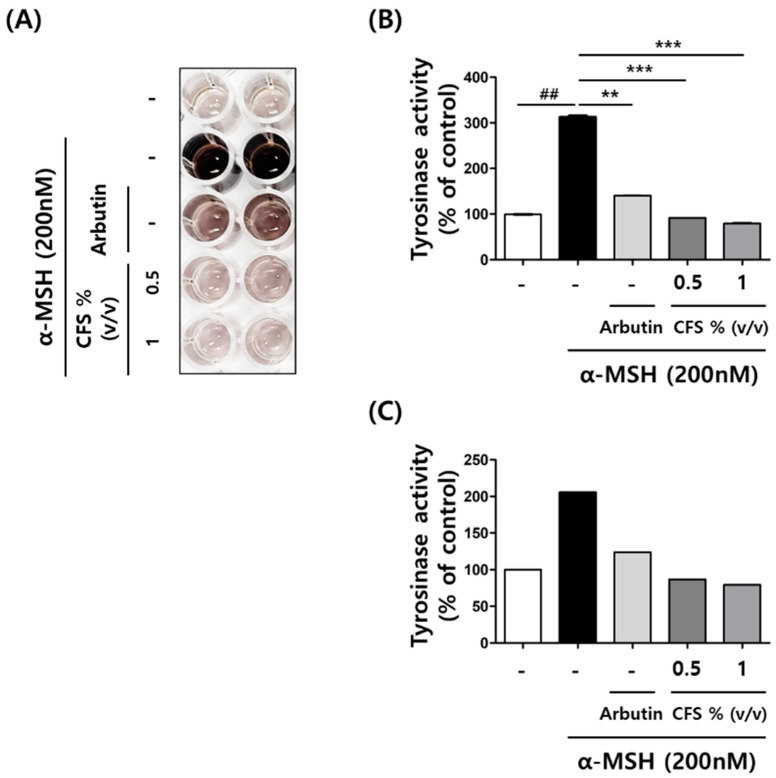
Effect of CFS on intracellular tyrosinase activity in B16-F10 cells. The tyrosinase activity levels were determined as described in the Materials and Methods. (**A**) The color of the tyrosinase activity after 40 h treatment of α-MSH, arbutin, and CFS. Intracellular tyrosinase activity was assayed using DOPA activity (**B**) and enzyme activity assay kit (**C**) in the extracted protein samples. CFS: cell-free supernatant from *L. gasseri* BNR17. Values represent the mean ± SD of three independent experiments. ^##^ *p* < 0.01 compared with control. ** *p* < 0.01 and *** *p* < 0.001 compared with the α-MSH-treated control.

Tyrosinase is an essential enzyme in melanin biosynthesis. It converts L-tyrosine to L-DOPA and then produces DOPA quinone, the precursor of melanin [20]. Therefore, we investigated the role of the CFS in intracellular tyrosinase activity in α-MSH-treated B16-F10 cells. In this experiment, arbutin was used as a positive control to evaluate the inhibition of enzyme activity on intracellular tyrosinase. As shown in Figure 4A, pretreatment with 0.5 and 1% (*v*/*v*) CFS brightened the colors of the culture media in a dose-dependent manner than that of only α-MSH-treated cells. For the analysis of intracellular tyrosinase activity, cell lysates pretreated with CFS were used as the intracellular enzyme sources of tyrosinases. The level of intracellular tyrosinase activities after only α-MSH treatment was 313%, which decreased to 91.5% and 79.5% upon pretreatment with 0.5 and 1% (*v*/*v*) CFS, respectively. The inhibitory activity of CFS was better than that of 200 µM arbutin, which decreased the intracellular tyrosinase activity to 140.4% (Figure 4B). These results were consistent with the data from the tyrosinase activity assay kit (Figure 4C), while CFS did not show any inhibition on the mushroom tyrosinase assay (data not shown).

### 2.4. CFS Downresgulates α-MSH-Induced Melanogenesis-Related Gene Expression in B16-F10

**Figure 5:**
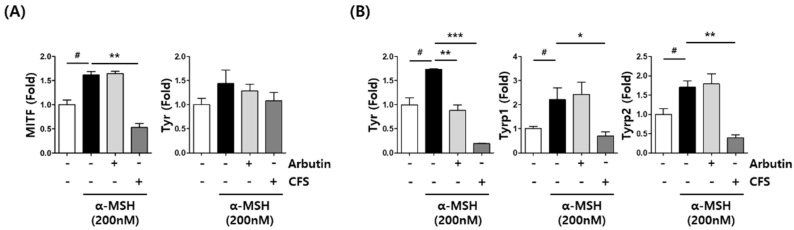
Effect of CFS on melanogenesis-related gene expression in B16-F10 cells. mRNA level of genes determined by real-time RT-PCR. (**A**) *Mitf* and *Tyr*, 6 h after treatment. (**B**) *Tyr, Tyrp1*, and *Tyrp2*, 40 h after treatment. B16-F10 cells were exposed to 200 nM α-MSH in the presence of 1% (*v*/*v*) CFS or 200 µM Arbutin. CFS: cell-free supernatant from *L. gasseri* BNR17. Values represent the mean ± SD of three independent experiments. ^#^ *p* < 0.05 compared with control. * *p* < 0.05, ** *p* < 0.01, and *** *p* < 0.001 compared with the α-MSH treated control.

Since CFS reduced melanin production by suppressing the intracellular tyrosinase activity, we next confirmed whether it affected the expression of melanogenesis-related genes, such as *Mitf*, *Tyr*, *Tyrp1*, and *Tyrp2*. Figure 5A demonstrates that the mRNA expression of Mitf significantly reduced after 1% (*v*/*v*) CFS treatment at 6 h. The mRNA levels of Mitf and Tyr were lower in the cells treated with CFS than in the cells treated with arbutin. Additionally, the high mRNA levels of *Tyr*, *Tyrp1*, and *Tyrp2* during α-MSH treatment significantly decreased by CFS treatment at 40 h (Figure 5B). These results suggested that CFS may modulate Mitf to decrease mRNA levels of melanogenesis-associated markers.

### 2.5. Radical Scavenging Activitiy of CFS

**Figure 6:**
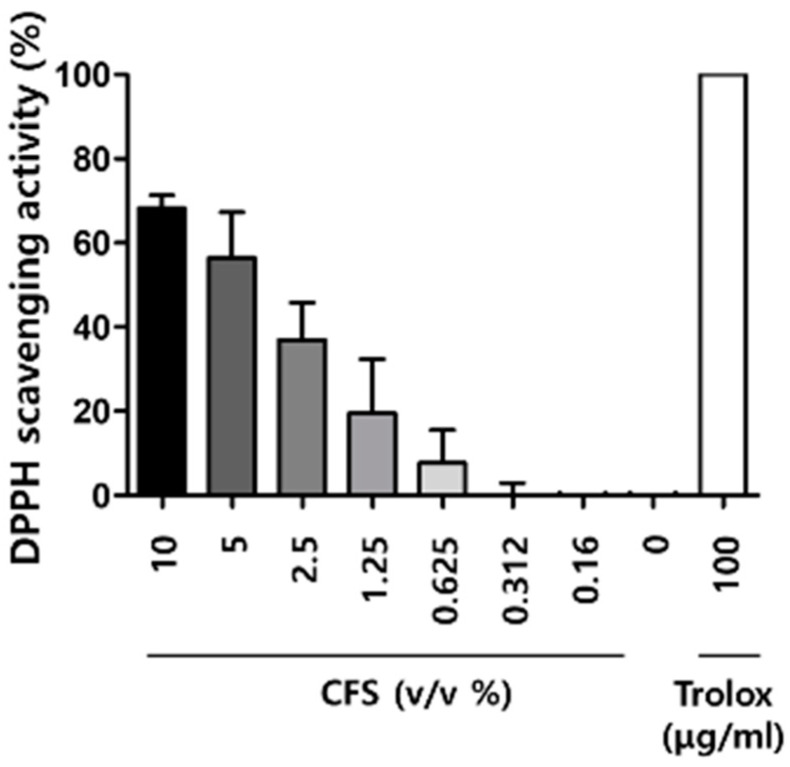
Effect of CFS on free radical scavenging activity by the DPPH assay. Trolox was used as positive control. CFS: cell-free supernatant from *L. gasseri* BNR17. Values represent the mean ± SD of three independent experiments.

ROS-induced oxidative stress results in melanin synthesis and skin damage [21,22]. Thus, antioxidants reduce melanin production and have skin-protecting properties. To investigate the antioxidative effect of CFS, we used various concentrations of the CFS (0.16, 0.312, 0.625, 1.25, 2.5, 5, and 10% (*v*/*v*)). Trolox (positive control) showed over 70% DPPH radical scavenging activity. The 2,2-diphenyl-1-picryl-hydrazyl-hydrate (DPPH) radical scavenging activities of the CFS increased in a dose-dependent manner. (Figure 6).

### 2.6. Effect of CFS on Antioxidant-Related Gene Expression in HaCaT Cells

**Figure 7:**
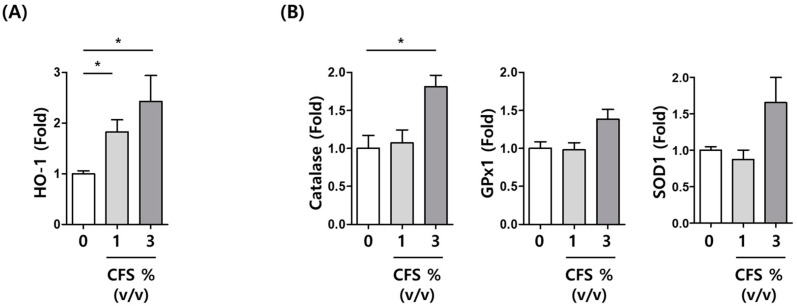
The expression levels of antioxidant-related gene after CFS treatment. The mRNA expression of (**A**) *HO-1* and (**B**) *CAT*, *GPX1*, and *SOD1* was determined by qRT-PCR. HaCaT cells were treated with CFS at a concentration of 1 or 3% (*v*/*v*) for 24 h. CFS: cell-free supernatant from *L. gasseri* BNR17. Data are presented as mean ± SEM. * *p <* 0.05 compared with the control.

To characterize the mechanisms involved in the antioxidative effect of CFS, we evaluated the effect of CFS on antioxidant-related gene expression. CFS treatment increased heme oxygenase 1 (HO-1) mRNA level in a concentration-dependent manner in HaCaT cells (Figure 7A). In line with this finding, CFS treatment significantly increased the mRNA levels of catalase (CAT), while glutathione peroxidase 1 (GPX1) and superoxide dismutase 1 (SOD1) mRNA showed a tendency to increase (Figure 4B). Thus, CFS elicits its antioxidative effect via inducing antioxidant-related gene expression.

**Figure 8:**
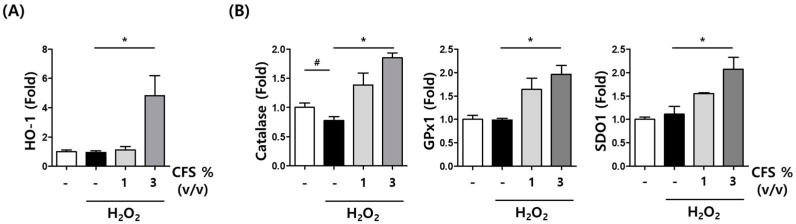
Antioxidant effects of CFS on H_2_O_2_-induced oxidative stress. HaCaT cells were preincubated with CFS at the concentration of 1 or 3% (*v*/*v*) for 30 min and then incubated with H_2_O_2_ (10 μM) for 24 h. qRT-PCR analysis for (**A**) *HO-1* and (**B**) *CAT*, *GPX1*, and *SOD1*. Data are presented as mean ± SEM. ^#^ *p* < 0.05 compared with control. * *p <* 0.05 compared with H_2_O_2_ treated group, respectively.

As mentioned earlier, oxidative stress is closely associated with melanin synthesis in melanocytes and skin damage in keratinocytes. During oxidative stress-induced skin damage, antioxidative and free radical scavenging activities are critical for skin protection. To investigate the intracellular antioxidative effect of CFS, HaCaT cells were pretreated with CFS (1 and 3% (*v*/*v*)) for 1 h, followed by 10 µM H_2_O_2_ for an additional 24 h, and the mRNA levels of *HO-1*, *CAT*, *GPX1*, and *SOD1* were measured using real-time reverse transcriptase PCR. Pretreatment with 3% (*v*/*v*) of CFS resulted in a dramatic increase in *HO-1*, *CAT*, GPX1, and *SOD1* mRNA levels, suggesting that the induction of antioxidant-related genes by CFS treatment is associated with skin protection (Figure 8A,B).

## 3. Discussion

Probiotic intake is known for its ability to optimize, maintain, and restore the skin’s microbiome in a variety of ways [23]. Additionally, several recent studies have reported that *Lactobacillus* may help improve skin health. As representative studies, *Limosilactobacillus fermentum* JNU532 and *Lactobacillus acidophilus* KCCM12625P were reported to be effective in antioxidant [24] and pigmentation inhibition [25], and *L. acidophilus* IDCC 3302 was reported to improve skin biological responses, such as antiphotodamage [26], anti-wrinkle [27], and skin moisturizing effect [28]. Despite these previously published studies, studies on the antioxidant and anti-pigmentation effects of *L. garssri* species as well as *L. garssri* BNR17 have not yet been conducted.

*Lactobacillus gasseri* BNR17 is isolated from human breast milk. It is widely used in weight management and improving post-menopausal symptoms. Although it is known to improve human health, the significance of CFS in skin protection has not been evaluated. Thus, we decided to investigate the benefits of CFS in skincare.

We found that CFS had a significant anti-melanogenic effect without cytotoxicity. The possible mechanisms of anti-melanogenesis were linked to the transcriptional regulation by transcription factors such as MITF to inhibit TYR, TYRP1, and TYRP2. In addition, it was associated with reducing tyrosinase activity. Further, CFS exerts its antioxidant effect via stimulating cytoprotective and anti-oxidant-related genes, such as *HO-1*, *CAT*, *GPX1*, and *SOD1*. In addition, CFS showed thermostability without any decrease in its anti-melanogenic efficacy. This finding agrees with the desirable criterion of any product to be adopted for industrial manufacture.

Dooley et al. [29] demonstrated that a skin lightening agent should inhibit melanin production in melanocytes by reducing the synthesis or activity of tyrosinase. In mammals, melanin is an essential biological pigment produced in melanocytes via three melanocyte-specific enzymes—TYR, TYRP1, and TYRP2. Tyrosinase initiates the melanin biosynthetic process by oxidizing tyrosine to L-DOPA, and TYRP-1 and TYRP-2 have been demonstrated to increase tyrosinase stability and induction of melanin synthesis [20]. In particular, MITF is a master transcription factor of the melanocyte lineage that stimulates melanogenesis by activating the transcription of tyrosinase, TYRP1, and TYRP2. In line with these previous studies, CFS appeared to have anti-melanogenic activity similar to that of the positive control arbutin in murine melanocytes. As illustrated in Figure 2, CFS significantly reduced melanin secretion and its intracellular accumulation in the presence of α-MSH, a major stimulator of melanin biosynthesis in melanocytes. Consistent with the data from α-MSH-induced melanin production, CFS not only reduced tyrosinase activity, but decreased the mRNA expression of *Mitf, Tyr, Tyrp1*, and *Tyrp2* in α-MSH-induced murine melanocytes, as well as showed thermostability with anti-melanogenic efficacy.

Antioxidants protect cells against oxidative stress-induced damage and are pivotal in the inhibition of melanogenesis in melanocytes and the maintenance of healthy keratinocytes [8,30]. The DPPH assay is widely used to measure the antioxidant properties in vitro. If a sample itself has antioxidant effects, it can remove free radicals. CFS exhibited antioxidant effects in the DPPH assay in vitro and in H_2_O_2_ treated-HaCaT cells (human keratinocytes) both by direct and indirect treatment. CFS treatment induced *HO-1* expression at the transcriptional levels. Interestingly, CFS pretreatment followed by H_2_O_2_ exposure increased *HO-1* expression more than that in the H_2_O_2_ treatment alone. Since *HO-1* protects cells from diverse cellular stress, such as oxidative stress, inflammation, and apoptotic cell death [31,32,33], increased levels of *HO-1* by CFS might have a crucial role in the relevant skin physiology. Further study will confirm additional skin functionality of CFS, such as anti-aging and moisturizing, and it is also necessary to identify the functional substances.

Since we did not investigate the component analysis from CFS of *L. gasseri* BNR17 in the analytical methods, we cannot fully explain the mechanism responsible for the anti-melanogenesis and antioxidant effects from CFS at present. To solve these problems, we are currently conducting amino acid analysis and HPLC analysis, and these analyses are in accordance with recently published reports. Various amino acids and peptides have anti-melanogenic properties by inhibiting tyrosinase activity or down-regulating TYR gene expression [34]. Additionally, proline-serine and valine-serine dipeptides are reported to have anti-melanogenic effects by down-regulating the expression of MITF and tyrosinase protein [35]. In the future, we plan to identify the components of CFS that affect anti-melanogenic and antioxidant effects by synthesizing the results of the further analysis. Moreover, additional research is needed to confirm the additional skin functionality of CFS, such as anti-aging and moisturizing.

## 4. Materials and Methods

### 4.1. Preparation of Cell-Free Culture Supernatant

*L. gasseri* BRN17 (Accession No: KCTC10902 BP) was isolated from the human breast milk and selected for its probiotic characteristics shown in a previous study [18]. *L. gasseri* BRN17 was aerobically cultured for 16 h at 37 °C according to previous studies [36]. Then, the CFS of the *L.* *gasseri* BNR17 cultured medium was transferred to 1.5 mL microcentrifuge tube and collected by centrifugation at 12,000× *g* for 10 min. Supernatant was transferred to a new microcentrifuge tube, and then it was filtered with a 0.22 μm pore size filter unit (Sartorius Stedim Biotech GmbH, Goettingen, Germany), subsequently. Thereafter, the CFS was stored at −20 °C until use. MRS broth medium (filtered with a 0.22 μm pore size filter) served as a negative control.

### 4.2. Cell Culture

The murine melanoma cell line B16-F10 (ATCC, Rockville, MD, USA) and the immortal human keratinocyte cell line HaCaT (ACCEGEN, Fairfield, NJ, USA) were grown in Dulbecco’s Modified Eagle’s Medium (WelGENE, Daegu, Korea) supplemented with 10% fetal bovine serum (HyClone, Logan, UT, USA) and antibiotic/antimycotic solution (HyClone) at 37 °C in a humidified incubator (5% CO_2_).

### 4.3. Chemicals and Reagents

Melanogenesis stimulator α-MSH and inhibitor arbutin were purchased from Sigma-Aldrich (St. Louis, MO, USA). TRIzol solution and bicinchoninic acid (BCA) protein assay kit were purchased from Thermo Fisher Scientific (Waltham, MA, USA).

### 4.4. Cell Viability Assay

Cell viability was determined by the WST assay (AbFrontier, Seoul, Korea). B16-F10 cells and HaCaT cells were plated into a 96-well plate at a density of 1 × 10^4^ cells/well for 24 h. In addition, to determine cell viability for 48 h, B16-F10 cells were plated into 96-well plate at a density of 5 × 10^3^ cells/well. After incubation, CFS was treated with various concentrations (0.1, 0.5, 1, 2.5, and 5% (*v*/*v*) or 1, 3, and 5% (*v*/*v*) for 24 h or 48 h at 37 °C in humidified air and 5% CO_2_). Following incubation, the cells were treated with WST for 2 h. The absorbance was measured at 450 nm using an EZ Read 800 microplate reader (Biochrom, Cambridge, UK). The percentage of cells showing cytotoxicity was determined relative to the control group.

### 4.5. Measurement of Melanin Content

The extracellular melanin content was measured using a slight modification of a previously reported method [37]. Briefly, B16-F10 cells were seeded in 6-well plates (1 × 10^5^ cells/well) and incubated for 24 h. After incubation, the culture media was replaced with the phenol-red free culture media containing CFS (0.5 and 1% (*v*/*v*)) or arbutin (200 µM) for 48 h in the presence or absence of 200 nM α-MSH. After treatment, we collected the media and measured the melanin levels at 492 nm using an ELISA reader. Intracellular melanin contents were determined following a modified method to assess extracellular melanin content. Briefly, B16-F10 cells were seeded in 6-well plates (1× 10^5^ cells/well) and incubated for 24 h. After incubation, the culture media was replaced with the phenol-red free culture media containing CFS (0.5 and 1% (*v*/*v*)) or arbutin (200 µM) for 48 h in the presence or absence of 200 nM α-MSH. The cells were collected by trypsinization and suspended in 150 µL of 1 N NaOH (Samchun Chemical, Seoul, Korea) in 10% dimethyl sulfoxide (Sigma-Aldrich). After heating at 60 °C for 2 h, the absorbance at 492 nm was measured using a microplate reader. The final relative melanin content was normalized to the cell numbers [38,39].

### 4.6. Analysis of Intracellular Tyrosinase Activity

The cellular tyrosinase activity were measured using tyrosinase Activity Assay Kit (Abcam, Cambridge, UK) and a slight modification of a previously reported method [40]. B16-F10 cells were seeded in 60 mm dishes (2 × 10^5^ cells/dish) for 24 h and treated with CFS (0.5 and 1% (*v*/*v*)) or arbutin (200 µM) for 40 h in the presence or absence of 200 nM α-MSH, harvested by trypsinization, sonicated in assay buffer (Abcam, Cambridge, UK), and centrifuged at 12,000 rpm for 20 min. The protein concentration was determined by the Pierce™ BCA Protein Assay Kit (Thermo Fischer Scientific). The reaction mixture consisting of 20 µg protein and 80 µL of 2 mg/mL L-DOPA (in 0.1 M sodium phosphate buffer, pH 6.8) was added to each well of a 96-well plate. After incubation at 37 °C for 1 h, the optical density at 492 nm was measured using a microplate reader.

### 4.7. Quantitative Reverse-Transcription Polymerase Chain Reaction

B16-F10 and HaCaT cells (5 × 10^4^ cells/well) were plated on 12-well plates and incubated. Then, B16-F10 cells were treated with 1% (*v*/*v*) CFS or arbutin (200 µM) for 6 h and 40 h in the presence or absence of 200 nM α-MSH. HaCaT cells were pretreated with CFS (1 and 3% (*v*/*v*)) for 30 min and then co-treated 100 µM H_2_O_2_ for 24 h. Then, the cells were harvested and washed twice with phosphate-buffered saline. Total cellular RNA was prepared using TRIzol solution according to the manufacturer’s instructions. RNA was converted to cDNA using RevertAid First Strand cDNA Synthesis Kit (Thermo Fisher Scientific), according to the manufacturer’s instructions. The 2X GreenStar qPCR Master Mix (Bioneer, Daejeon, Korea) was used in all the samples, and reactions were carried out in a 20 µL final reaction volume. Each experiment was performed at least twice in duplicates using the following primers in Supplementary Table S1. All gene expression levels were calculated by using the Ct value by the method 2^−ΔΔCt^ (where ΔCt = Ct_[target gene]_ − Ct_[GAPDH]_).

### 4.8. DPPH Assay

The antioxidative effect of CFS was determined with OxiTec™ DPPH Antioxidant Assay Kit (BIOMAX, Seoul, Korea) according to the manufacturer’s instructions. Briefly, the various concentration of CFS was prepared, and the DPPH working solution and assay buffer were individually added into a 96-well assay plate. The plate was incubated at room temperature for 30 min in the dark, and the absorbance was measured at 517 nm using a microplate reader. The superoxide anion radical scavenging activity of CFS samples was evaluated by Trolox (a water-soluble analog of vitamin E) standard. The superoxide anion radical scavenging activity of CFS samples was normalized to the scavenging activity of 100 μg/mL Trolox.

### 4.9. Statistical Analysis

All data were analyzed using GraphPad Prism 5 software (La Jolla, CA, USA). Analyses were performed using the Student’s *t*-test or the one-way analysis of variance test for multiple comparisons. For all comparisons, *p* < 0.05 was considered statistically significant.

## 5. Conclusions

In summary, our present study suggests the potential of *L. gasseri* BNR17-derived CFS in improving skin health. CFS inhibits melanogenesis while maintaining thermal stability by downregulating transcription factors, melanogenesis-related genes, and tyrosinase activity. Furthermore, CFS was shown to have antioxidant properties that could inhibit melanin production and oxidative-stress-induced cell damage. Based on these findings, the cell-free supernatant of *L. gasseri* BNR17 could be developed as a promising product for skincare.

## Data Availability

Not applicable.

## Supplementary Materials

The following supporting information can be downloaded at: https://www.mdpi.com/article/10.3390/microorganisms10040788/s1, Figure S1: The thermostability of CFS on melanogenesis in B16-F10, Table S1: Primer information.
