# Supplementary material for: Anti-Melanogenic and Antioxidant Effects of Cell-Free Supernatant from Lactobacillus gasseri BNR17
Source: Microorganisms. 2022 Apr 8;10(4):788. doi: 10.3390/microorganisms10040788 (PMC9027439; doi:10.3390/microorganisms10040788)
Supplement: Supplementary file 1 [file microorganisms-10-00788-s001.zip › microorganisms-1643522-supplementary.pdf]

Anti-melanogenic and antioxidant effects of cell-free supernatant from *Lactobacillus gasseri* BNR17

Sol Lee[1,2], Han-Oh Park[1,2,3,4], Wonbeak Yoo[1,2,*]

1 AceBiome Inc., Seoul 06164, Republic of Korea
2 R&D Center, AceBiome Inc., Daejeon 34013, Republic of Korea
3 siRNAgen Therapeutics, Daejeon, 34302, Republic of Korea
4 Bioneer Corporation, Daejeon 34302, Republic of Korea
* Correspondence: wbyoo@acebiome.com; Tel.: (+82-42-335-6020)

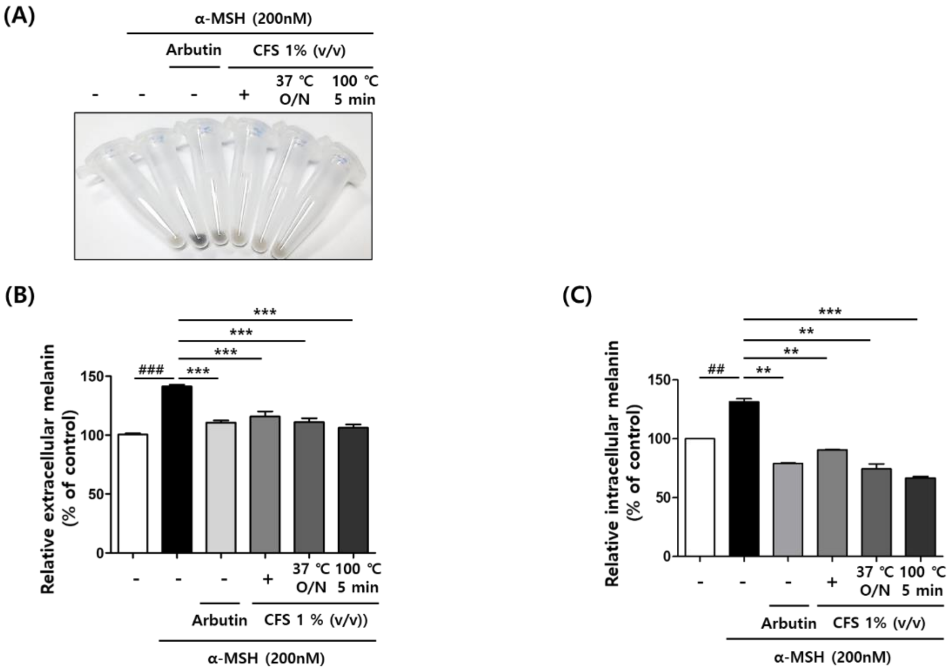

Figure S1. The thermostability of CFS on melanogenesis in B16-F10. The thermostability of melanogenesis inhibitory action of the CFS were checked. The CFS were made non-heated, 37 °C for overnight (O/N) and 100 °C for 5 min to check the thermostability, respectively, and checked for the activity of the anti-melanogenesis. CFS: cell-free supernatant from *L. gasseri* BNR17. Data are presented as mean + SD. $^{\#\#}$ $p < 0.01$ and $^{\#\#\#}$ $p < 0.001$ compared with control. ** $p < 0.01$ and *** $p < 0.01$ compared with the α-MSH treated control, respectively.

Materials and method

To determine the thermostability of CFS, CFS samples were prepared non-heated or heat 37 °C for overnight (O/N) and 100 °C for 5 min. All the prepared CFS samples were treated after filtration with 0.22 μm filter (Sartorius, Göttingen, Germany). The measurement of extracellular and intracellular melanin content was performed by the same method as described in the current manuscript.

Table S1. Primer information

Gene	Forward (5'-3')	Reverse (5'-3')
**Mouse**		
Mitf	AGGACCTTGAAAACCGACAG	GTGGATGGGATAAGGGAAAG
Tyr	AGCCTGTGCCTCCTCTAA	AGGAACCTCTGCCTGAAA
Tyrp1	CGATACCCTGGGAACACT	TACACGGACCTCCAAGCA
Tyrp2	CCAACGCTGATTAGTCGGA	GAAGAAGGGAGGGCTGTCA
Gapdh	GCCAAACGGGTCATCATCTC	GTCATGAGCCCTTCCACAAT
**Human**		
HO-1	AACTTTCAGAAGGGCCAGGT	CTGGGCTCTCCTTGTTGC
Catalase	ACTTTGAGGTCACACATGACATT	CTGAACCCGATTCTCCAGCA
GPx1	ACACCCAGATGAACGAGCTG	CAAACTGGTTGCACGGGAAG
SOD1	ATGACTTGGGCAAAGGTGGA	GGGCGATCCCAATTACACCA
GAPDH	CCACTCCTCCACCTTTGAC	ACCCTGTTGCTGTAGCCA
